# Polypharmacy and potential drug–drug interactions in emergency department patients in the Caribbean

**DOI:** 10.1007/s11096-017-0520-9

**Published:** 2017-08-09

**Authors:** Darren Dookeeram, Satesh Bidaisee, Joanne F. Paul, Paula Nunes, Paula Robertson, Vidya Ramcharitar Maharaj, Ian Sammy

**Affiliations:** 1 Eastern Regional Health Authority of Trinidad and Tobago, Sangre Grande Regional Hospital, Ojoe Road, Sangre Grande, Trinidad and Tobago; 2 St George’s University, True Blue, Grenada; 3 Faculty of Medical Sciences, The University of the West Indies, St Augustine Eric Williams Medical Sciences Complex, Champs Fleurs, Trinidad and Tobago; 4 North Central Regional Health Authority, Eric Williams Medical Sciences Complex, Champs Fleurs, Trinidad and Tobago; 5 School of Health and Related Research, University of Sheffield, Regent Court, 30 Regent Street, Sheffield, S1 4DA UK

**Keywords:** Aged, Drug interactions, Emergency Service, Hospital, Herb-Drug Interactions, Low-and-middle-income country, Trinidad and Tobago

## Abstract

*Background* Potential Drug–Drug Interactions (DDI) account for many emergency department visits. Polypharmacy, as well as herbal, over-the-counter (OTC) and combination medication may compound this, but these problems are not well researched in low-and-middle-income countries. *Objective* To compare the incidence of drug–drug interactions and polypharmacy in older and younger patients attending the Emergency Department (ED). *Setting* The adult ED of a tertiary teaching hospital in Trinidad. *Methods* A 4 month cross sectional study was conducted, comparing potential DDI in older and younger patients discharged from the ED, as defined using Micromedex 2.0. *Main outcome measure* The incidence and severity of DDI and polypharmacy (defined as the use of ≥5 drugs simultaneously) in older and younger patients attending the ED. *Results* 649 patients were included; 275 (42.3%) were ≥65 years and 381 (58.7%) were female. There were 814 DDIs, of which 6 (.7%) were contraindications and 148 (18.2%) were severe. Polypharmacy was identified in 244 (37.6%) patients. Older patients were more likely to have potential DDI (67.5 vs 48.9%) and polypharmacy (56 vs 24.1%). Herbal products, OTC and combination drugs were present in 8, 36.7 and 22.2% of patients, respectively. On multivariate analysis, polypharmacy and the presence of hypertension and ischaemic heart disease were associated with an increased risk of potential DDI. *Conclusion* Polypharmacy and potential drug–drug interactions are common in ED patients in the Caribbean. Older patients are particularly at risk, especially as they are more likely to be on multiple medications. The association between herbal medication and polypharmacy needs further investigation. This study indicates the need for a more robust system of drug reconciliation in the Caribbean.

**Electronic supplementary material:**

The online version of this article (doi:10.1007/s11096-017-0520-9) contains supplementary material, which is available to authorized users.

## Impact on practice

- Polypharmacy and potential drug–drug interactions are common in Emergency Department patients in Trinidad.
- The incidence of polypharmacy and potential drug–drug interaction problems is significantly higher in older patients
- Potential drug–drug interactions are independently associated with polypharmacy and the presence of hypertension and ischaemic heart disease

## Introduction

The prevalence of drug–drug interactions (DDI) involving prescription drugs in Emergency Department patients in the Caribbean is not known. However, research from other developing countries suggests that polypharmacy and drug–drug interactions might also be common in the Caribbean. Andreazza et al. [1] studied Emergency Department (ED) patients in Brazil; one in three presented with drug-related problems, including drug–drug interactions, adverse drug reactions and allergic drug reactions. Al-Olah in Saudi Arabia further concluded that 83% of the adverse drug effects seen in Emergency Department patients were avoidable [2]. While the demographics of some of these countries, such as Brazil, may be similar to that seen in the West Indies, it is unclear whether health behaviour and prescribing are comparable [1, 3].

Polypharmacy has been identified as the single most important risk factor for DDI [3]. There is no universally agreed definition of polypharmacy, but many researchers describe it as the concurrent use of 5 or more medications [4]. The risks of polypharmacy and DDI are higher among older people, who often suffer from a variety of medical conditions and are thus prescribed multiple medications, some of which may interact with each other to produce unwanted effects. For example, Ruiter et al. [5] found a four-fold increase in the risk of hospitalisation from adverse drug reactions and DDIs in patients aged 75 and older compared to those aged 55–74 years.

Researchers have demonstrated an association between DDI, polypharmacy and acute hospital admission. A systematic review of DDI in the ED conducted by Becker et al. [6] concluded that polypharmacy and DDIs remained a significant cause of morbidity, particularly among older patients. For example, Spaniolas et al. [7] showed that older trauma patients presenting to the ED with falls were more likely to be on multiple medications when compared to other older trauma patients. Drug-induced cognitive impairment, which may be exacerbated by drug–drug interactions, is another important cause of morbidity in older patients. This increases the chance of hospital admission while worsening outcome [8].

Reports from Jamaica have highlighted the relatively high prevalence of herbal medication use among hospital patients and those with chronic illnesses, but we could not find any Caribbean studies on polypharmacy and DDI in Emergency Department patients [9, 10]. Our study, therefore, sought to investigate medication use, polypharmacy and DDI in adults attending the ED of a large tertiary hospital in Trinidad and Tobago, comparing older and younger patients.

## Aims of the study

The main aim of this cross-sectional study was to assess the proportion of adult patients discharged from the ED of a tertiary teaching hospital in Trinidad and Tobago with polypharmacy and potential DDI, comparing patients aged 18–64 years with those aged ≥65 years. Secondary objectives were to determine the association of key demographic and clinical variables (including the use of herbal medication) with polypharmacy and potential DDI.

## Ethics approval

Ethical approval for this study was obtained from the research ethics committee of the Faculty of Medical Science, the University of the West Indies. Informed consent was obtained in writing from each study participant prior to data collection.

## Methods

Data were collected prospectively over a 4 month period in the ED of a tertiary teaching hospital. A convenience sampling method was used, but patients were recruited during all shifts (including night shifts) and on all days of the week (including weekends). Data were collected using a data collection sheet designed for the study. The main outcomes were the presence of polypharmacy [defined as the use of 5 or more medications, including over-the-counter drugs (OTCs)], and the presence of potential DDI as defined using Micromedex 2.0. When assessing polypharmacy, OTC medications were included, and each constituent active ingredient of any combination therapy was counted separately. However, herbal remedies were not counted.

All patients aged ≥18 years old with a Canadian Triage Acuity Scale (CTAS) category of 2–5, who were discharged from the ED after assessment and treatment, were eligible for inclusion in the study. We focused on patients being discharged from hospital as these were thought to be particularly at risk; patients admitted to hospital have the opportunity to have their medications reviewed by the inpatient team, but discharged patients may not have any further opportunity to have their potential drug interactions identified and corrected, thus putting them at higher risk of an adverse drug event.

Patients were excluded from the study if they were admitted to hospital or were too unwell to participate (including patients undergoing active resuscitation and those too confused to participate). Patients who consented were divided into two age cohorts for comparison (18–64 years and 65 years and older). The minimum sample size was calculated as 276 subjects per study group (a total of 552 patients), anticipating an incidence of potential DDI of 25% (based on data from previous research), and accepting a margin of error of 5%. The prevalence of DDI was estimated from studies from similar (low-and-middle-income country) settings as in Trinidad. It is not clear whether prescribing and health-seeking behaviours would have been similar in these populations, but we considered these setting the best approximation to what was likely to be found in our study [3, 11].

Data were analysed for statistical significance using SPSS version 21 (IBM Statistics, New York). Chi squared analysis (or Fishers Exact Test) was used to compare the prevalence of polypharmacy and potential DDI between older and younger patients, using a confidence level of 95% and a *p* value of .05. Logistic regression was used to determine the association between specific covariates and these outcomes. Covariates included in the regression model were age; gender; ethnicity; education level; use of over the counter (OTC) medication, herbal medication or combined medication; polypharmacy; and the presence of chronic illnesses (diabetes mellitus, hypertension, ischaemic heart disease, psychiatric illness and asthma). “Combination medication” refers to combinations which could include prescription and/or OTC formulations, but did not include herbal remedies. Univariate analysis was initially performed to determine the association between each covariate and the outcome of interest (either polypharmacy or potential DDI). Polypharmacy was included as a covariate in the regression analysis for DDI. Covariates with a moderate association with each outcome (*p* value <.2) were included in the multivariate model. For the multivariate model, a *p* value of < .05 was considered as statistically significant.

## Results

There were 649 patients in the study, of which 275 were aged ≥65 years and 374 were 18–64 years old. 268 (41.3%) patients were male, and 381 (58.7%) female. The ethnicity and educational levels of the respondents are shown in Table 1.

**Table 1:** Demographic and clinical characteristics of the study population

Total		Age ≥65 years	Age 18–64 years	All patients	*p* value
		275	374	649	
Gender					
Male	n (%)	119 (43.3%)	149 (39.8%)	268 (41.3%)	.42
Female	n (%)	156 (56.7%)	225 (60.2%)	381 (58.7%)	
Ethnicity					
Indo-trinidadian	n (%)	119 (43.3%)	129 (34.5%)	248 (38.2%)	.121
Afro-trinidadian	n (%)	105 (38.2%)	173 (46.3%)	278 (42.8%)	
Mixed	n (%)	49 (17.8%)	68 (18.2%)	117 (18%)	
Other	n (%)	2 (.7%)	4 (1.1%)	6 (.9%)	
Education					
None	n (%)	16 (5.8%)	8 (2.1%)	24 (3.7%)	<.001
Primary	n (%)	130 (47.3%)	88 (23.5%)	218 (33.6%)	
Secondary	n (%)	104 (37.8%)	208 (55.6%)	312 (48.1%)	
Vocational	n (%)	5 (1.8%)	15 (4%)	20 (3.1%)	
University	n (%)	19 (6.9%)	52 (13.9%)	71 (10.9%)	
Not recorded	n (%)	1 (.4%)	3 (.8%)	4 (.6%)	
Medical conditions					
DM	n (%)	121 (44%)	46 (12.3%)	167 (25.7%)	<.001
HTN	n (%)	153 (55.6%)	74 (19.8%)	227 (35%)	<.001
IHD	n (%)	54 (19.6%)	14 (3.7%)	68 (10.5%)	<.001
Psych	n (%)	27 (9.8%)	7 (1.9%)	34 (5.2%)	<.001
Asthma	n (%)	18 (6.5%)	39 (10.4%)	57 (8.8%)	.055

*DM* diabetes mellitus, *HTN* hypertension, *IHD* ischaemic heart disease, *Psych* psychiatric illness

Polypharmacy was more common in older patients, with 154 (56%) taking 5 or more medications compared to 90 (24.1%) younger adults (*p* < .001). Over the counter medication was used by 238 (36.7%) patients, while 52 (8%) admitted to using herbal remedies, and there was no significant difference in the use of either with age (*p* = .934 for OTCs and *p* = .306 for herbal remedies). Combination drugs were used in 144 (22.2%) patients, with a higher proportion of older people using these preparations (25.1 vs 22.2%, *p* = .036). The specific herbal and combination therapies reported by respondents are shown in Appendix 1 and 2 (ESM), respectively (Table 2).

**Table 2:** Medication usage and frequency of potential drug–drug interactions

Total		Age ≥65 years	Age 18–64 years	All patients	*p* value
		275	374	649	
Medication usage patterns					
OTC	n (%)	100 (36.4%)	138 (36.9%)	238 (36.7%)	.934
Herbal	n (%)	18 (6.5%)	34 (9.1%)	52 (8%)	.306
Combine	n (%)	50 (18.2%)	94 (25.1%)	144 (22.2%)	.036
Polypharmacy	n (%)	154 (56%)	90 (24.1%)	244 (37.6%)	<.001
Number of potential interactions seen in each patient					<.001
0	n (%)	92 (33.5%)	191 (51.1%)	283 (43.6%)	<.001
1–5	n (%)	53 (19.3%)	57 (15.2%)	110 (16.9%)	.162
6–10	n (%)	73 (26.5%)	37 (9.9%)	110 (16.9%)	<.001
>10	n (%)	35 (12.7%)	6 (1.6%)	41 (6.3%)	<.001
Patients on <2 drugs	n (%)	22 (8.0%)	83 (22.2%)	105 (16.2%)	<.001
Most severe potential interaction seen in each patient					<.001*
Contraindication	n (%)	6 (3.7%)	0 (0%)	.430	.430
Potential major interaction	n (%)	62 (38.5%)	24 (22%)	86 (33.0%)	.005
Potential moderate interaction	n (%)	88 (54.7%)	66 (66%)	154 (59%)	.070
Potential minor interaction	n (%)	5 (3.1%)	10 (10%)	15 (5.7%)	.020
Total	n (%)	161 (100%)	100 (100%)	261 (100%)	

* Significance calculated using fisher exact test with post hoc analysis

*OTC* over the counter

Of the 544 patients on two or more medications, 261 (48.0%) had at least one potential drug–drug interaction, including decreased drug efficacy (due to inhibition of absorption and distribution, as well as enhanced metabolism); enhanced drug effects (due to enhanced absorption, increased bioavailability and decreased metabolism and excretion) and an increased risk of gastrointestinal (GI) haemorrhage, bleeding (from sites other than the GI tract), hypotension, thrombosis, hypoglycaemia, hyperglycaemia, rhabdomyolysis, hyperkalaemia and lactic acidosis (Table 3). There was a significant increase in the number of potential DDI seen in older patients compared to younger patients (Table 2). Of the 261 patients with potential DDI, there were 6 (2.3%) patients on combinations of drugs that were contraindicated. The number of patients with potential major, moderate and minor interactions as their most serious potential DDI was 84 (32.2%), 154 (59%) and 15 (5.7%) respectively. Older patients were significantly more likely to have more serious interactions compared to their younger counterparts (Table 2).

**Table 3:** Characteristics of potential drug–drug interactions

Drugs involved		Severity of interaction (only commonest drugs listed)				Total
		Contra-indications	Potential major interaction	Potential moderate interaction	Potential minor interaction	
Aspirin	n (%)	0 (0%)	34 (23%)	236 (39.7%)	11 (16.4%)	281 (34.4%)
Lisinopril	n (%)	0 (0%)	6 (4.1%)	99 (16.6%)	9 (13.4%)	114 (14.0%)
Clopidogrel	n (%)	0 (0%)	2 (1.4%)	80 (13.4%)	1 (1.5%)	83 (10.2%)
Enalapril	n (%)	0 (0%)	1 (.7%)	62 (10.4%)	0 (0%)	63 (7.7%)
Atenolol	n (%)	0 (0%)	2 (1.4%)	60 (10.1%)	0 (0%)	62 (7.6%)
Bendroflumethiazide	n (%)	0 (0%)	53 (35.8%)	8 (1.3%)	0 (0%)	61 (7.5%)
Glipizide	n (%)	3 (50%)	8 (5.4%)	32 (5.4%)	18 (26.9%)	61 (7.5%)
Diclofenac	n (%)	0 (0%)	2 (1.4%)	58 (9.7%)	0 (0%)	60 (7.4%)
Metformin	n (%)	0 (0%)	5 (3.4%)	38 (6.4%)	13 (19.4%)	56 (6.9%)
Nifedipine	n (%)	0 (0%)	4 (2.7%)	46 (7.7%)	1 (1.5%)	51 (6.3%)
*Type of Interaction(only commonest interactions listed)*						
Decreased efficacy	n (%)	2 (33.3%)	25 (16.9%)	291 (48.9%)	38 (56.7%)	356 (43.6%)
Enhanced effects	n (%)	2 (33.3%)	18 (12.2%)	156 (26.2%)	18 (26.9%)	194 (23.8%)
Hypoglycaemia	n (%)	0	11 (7.4%)	82 (13.8%)	1 (1.5%)	94 (11.5%)
Hypotension	n (%)	0	4 (2.7%)	70 (11.8%)	0 (.0%)	74 (9.1%)
Hyerglycemia	n (%)	0	11 (7.4%)	39 (6.6%)	0 (.0%)	50 (6.1%)
Rhabdomyolysis	n (%)	1 (16.7%)	33 (22.4%)	1 (.2%)	0 (.0%)	35 (4.3%)
GI hemorrhage	n (%)	0	0 (.0%)	25 (4.2%)	9 (13.4%)	34 (4.2%)
Bleeding (other)	n (%)	0	62 (41.9%)	28 (4.7%)	1 (1.5%)	91 (11.2%)
Thrombosis	n (%)	0	22 (14.8%)	2 (3.3%)	0 (.0%)	24 (2.9%)
Hyperkalemia	n (%)	0	6 (4.1%)	10 (1.7%)	0 (.0%)	16 (2.0%)
Lactic acidosis	n (%)	0	0 (.0%)	16 (2.7%)	0 (.0%)	16 (2.0%)
Total	n (%)	6 (100%)	148 (100%)	595 (100%)	67 (100%)	814 (100%)

Only the commonest drugs and interactions are listed

*GI* gastro-intestinal

In total, there were 814 potential DDIs in the 649 patients included in the study. The drugs most commonly implicated were aspirin (281 interactions), lisinopril (114 interactions) and clopidogrel (83 interactions). The other drugs commonly implicated in potential DDI are listed in Table 3. Glipizide was listed in three [3] of the six contraindications identified. Bendrofluazide was the drug most commonly involved in major potential interactions, while aspirin was the drug most commonly implicated in moderate potential interactions.

The multivariate logistic models for predicting polypharmacy and potential DDI are shown in Table 4 and 5 respectively. The variables associated with an increased risk of polypharmacy on multivariate analysis were age, the use of OTC, herbal and combined medications, and the presence of hypertension, ischaemic heart disease and psychiatric illness. On univariate analysis, polypharmacy was significantly more likely in female patients and those with no formal education, but these associations did not persist in the multivariate model. The variables associated with an increased risk of potential drug–drug interaction on multivariate analysis were polypharmacy and the presence of hypertension and ischaemic heart disease. Asthma and the use of combination therapies were both associated with a decreased risk of potential DDI. The risk of potential DDI was not influenced by age, herbal medication use or OTC use. However, it should be noted that Micromedex 2.0 may not have identified all potential DDIs associated with herbal formulations.

**Table 4:** Multivariate logistic regression analysis of factors associated with polypharmacy

	No. of patients (%)		Univariate analysis				Multivariate analysis			
	No Polypharmacy	Polypharmacy	Unadjusted odds ratio	95% C.I.		*p* value	Adjusted odds ratio	95% C.I.		*p* value
				Lower	Upper			Lower	Upper	
Age										
18–64	284 (75.9%)	90 (24.1%)	Reference							
≥65	121 (44%)	154 (56%)	4.016	2.870	5.620	<.001	2.373	1.435	3.923	.001
Gender										
Male	180 (67.2%)	88 (32.8%)	Reference							
Female	225 (59.1%)	156 (40.9%)	1.418	1.023	1.966	.036	.992	.624	1.576	.972
Ethnicity										
Indo-trinidadian	146 (58.9%)	102 (41.1%)	Reference							
Afro-trinidadian	181 (65.1%)	97 (34.9%)	.767	.539	1.092	.141				
Mixed	74 (63.2%)	43 (36.8%)	.832	.529	1.308	.425				
Other	4 (66.7%)	2 (33.3%)	.716	.129	3.981	.702				
Education										
None	6 (25%)	18 (75%)	Reference							
Primary	118 (54.1%)	100 (45.9%)	.282	.108	.739	.010	.297	.075	1.174	.083
Secondary	217 (69.6%)	95 (30.4%)	.146	.056	.379	<.001	.314	.081	1.221	.095
Vocational	11 (55%)	9 (45%)	.273	.076	.978	.046	1.196	.209	6.834	.840
University	51 (71.8%)	20 (28.2%)	.131	.045	.377	.000	.255	.057	1.145	.075
Not recorded	2 (50%)	2 (50%)	.333	.038	2.910	.320	2.535	.106	60.427	.565
Medical formulation										
OTC	107 (45%)	131 (55%)	3.229	2.310	4.513	<.001	5.243	3.212	8.559	<.001
Herbal	25 (48.1%)	27 (51.9%)	1.891	1.071	3.341	.028	3.608	1.649	7.896	.001
Combination	63 (43.8%)	81 (56.3%)	2.698	1.848	3.938	<.001	7.590	4.317	13.347	<.001
Chronic illness										
DM	55 (32.9%)	112 (67.1%)	5.399	3.693	7.894	<.001	4.499	2.589	7.817	<.001
HTN	68 (30%)	159 (70%)	9.270	6.400	13.427	<.001	7.227	4.316	12.101	<.001
IHD	10 (14.7%)	58 (85.3%)	12.317	6.157	24.640	<.001	7.774	3.294	18.348	<.001
Psych	7 (20.6%)	27 (79.4%)	7.074	3.031	16.512	<.001	4.341	1.428	13.201	.010
Asthma	32 (56.1%)	25 (43.9%)	1.331	.768	2.304	.308				

Each ‘medical formulation’ and ‘chronic illness’ was treated as an independent covariate in the equation; for each of these variables, patients without the variable were used as the reference

*OTC* over the counter, *DM* diabetes mellitus, *HTN* hypertension, *IHD* ischaemic heart disease

**Table 5:** Multivariate logistic regression analysis of factors associated with potential drug–drug interactions (DDI)

	No. of patients (%)		Univariate analysis				Multivariate analysis			
	No potential DDI	Potential DDI	Unadjusted odds ratio	95% C.I.		*p* value	Adjusted odds ratio	95% C.I.		*p* value
				Lower	Upper			Lower	Upper	
Age										
18–64	191 (65.6%)	100 (34.4%)	Reference							
>=65	92 (36.4%)	161 (63.6%)	3.342	2.350	4.754	<.001	1.222	.758	1.972	.409
Gender										
Male	122 (56.7%)	93 (43.3%)	Reference							
Female	161 (48.9%)	168 (51.1%)	1.369	.969	1.934	.075	1.543	.987	2.412	.057
Ethnicity										
Indo-trinidadian	109 (51.9%)	101 (48.1%)	Reference							
Afro-trinidadian	118 (51.3%)	112 (48.7%)	1.024	.704	1.489	.900				
Mixed	54 (53.5%)	47 (46.5%)	.939	.584	1.511	.796				
Other	2 (66.7%)	1 (33.3%)	.540	.048	6.042	.617				
Education										
None	7 (30.4%)	16 (69.6%)	Reference							
Primary	82 (42.3%)	112 (57.7%)	.598	.235	1.519	.279	.869	.269	2.814	.815
Secondary	153 (60.5%)	100 (39.5%)	.286	.114	.720	.008	.717	.224	2.298	.575
Vocational	10 (58.8%)	7 (41.2%)	.306	.082	1.137	.077	.886	.176	4.452	.883
University	30 (54.5%)	25 (45.5%)	.365	.130	1.026	.056	1.329	.362	4.881	.668
Not recorded	1 (50%)	1 (50%)	.438	.024	8.036	.578	.281	.004	20.309	.561
Medical formulation										
OTC	102 (47.7%)	112 (52.3%)	1.334	.945	1.883	.102	.790	.485	1.288	.345
Herbal	28 (56%)	22 (44%)	.838	.467	1.506	.555	.710	.320	1.577	.401
Combination	83 (58.5%)	59 (41.5%)	.704	.478	1.036	.075	.533	.309	.919	.024
Polypharmacy	59 (24.2%)	185 (75.8%)	9.242	6.245	13.676	<.001	6.392	3.691	11.069	.000
Chronic illness										
DM	45 (27.8%)	117 (72.2%)	4.297	2.877	6.419	<.001	1.442	.842	2.469	.182
HTN	47 (21.3%)	174 (78.7%)	10.043	6.696	15.061	<.001	3.972	2.437	6.473	<.001
IHD	8 (11.8%)	60 (88.2%)	10.261	4.800	21.937	<.001	3.633	1.477	8.938	.005
Psych	6 (18.2%)	27 (81.8%)	5.327	2.162	13.123	<.001	2.514	.851	7.429	.095
Asthma	35 (71.4%)	14 (28.6%)	.402	.211	.765	<.001	.350	.152	.807	.014

Each ‘medical formulation’ and ‘chronic illness’ was treated as an independent covariate in the equation; for each of these variables, patients without the variable were used as the reference

*OTC* over the counter, *DM* diabetes mellitus, *HTN* hypertension, *IHD* ischaemic heart disease

## Discussion

This study revealed a high percentage of patients with potential drug–drug interactions, and many with multiple potential interactions. Both polypharmacy and potential DDI were more common in older patients (aged ≥65 years), and there was an association between polypharmacy and the use of herbal and over-the-counter medications. In addition, potential DDI was significantly associated with polypharmacy and certain common chronic diseases (including hypertension, ischaemic heart disease and psychiatric conditions).

The relationship between potential DDI, polypharmacy and increasing age is well recognised in the research literature, and our study suggests that these associations are also seen in Caribbean patients. Banerjee et al. [4] noted the high rate of polypharmacy in older ED attenders in the United Kingdom, while Hovstadius [12] (Sweden, 2006) found a strong positive correlation between polypharmacy and older age. Our study helps to shed some light on the nature of the relationship between age and potential DDI: while older people were found to have a higher incidence of potential DDI, there was no independent association between age and potential DDI on multivariate analysis. However, increasing age has been associated with polypharmacy, chronic illness and female gender in the past, and all of these variables have also been found to be associated with DDI in previous studies [12, 13].

Our study also highlighted a significant association between the use of over-the-counter medication, combination therapies and herbal medication and the presence of polypharmacy. In our multivariate model, the use of OTC medication was associated with a five-fold increase in the adjusted odds of polypharmacy, while patients on combination therapies had a seven-fold increase. For patients using herbal medication, the adjusted odds of polypharmacy were three times that of other patients. In the Caribbean, a drug history should therefore include specific questions about herbal, OTC and combination therapies.

Interestingly, there was no significant association between the use of OTC, herbal or combination therapies and the risk of potential DDI. In fact, on multivariate analysis, the odds of potential DDI in patients using combination therapies was actually less than for other patients. It is not clear why the association between these therapies and polypharmacy did not translate to an increased risk of potential DDI. This may have been due to the confounding effects of other demographic variables, such as age and the presence of chronic illnesses. It should be noted, however, that our study was not specifically powered to detect any differences in potential DDIs in patients on OTCs, combination therapies or herbal formulations. As discussed earlier, previous work in Jamaica highlighted the association between herbal medication, polypharmacy and potential DDI in diabetic patients [9, 10]. Similar associations may also exist in other patients with chronic illnesses, such as ischaemic heart disease and hypertension.

The significant association between common chronic illnesses such as ischaemic heart disease, hypertension and diabetes and potential DDI is not unexpected, given that these patients were more likely to be on multiple drugs. In addition to this, 11 of the top 12 drugs most commonly implicated in potential DDI were prescribed for these conditions. Of these drugs, aspirin, bendrofluazide, glipizide, lisinopril and diltiazem accounted for the majority of major potential interactions and contraindications. This emphasizes the importance of drug reconciliation in patients with common chronic illnesses who present to the Emergency Department. In contrast, it is likely that the decrease in potential DDI with asthma was because the drug combinations used in asthma follow the GINA (Global Initiative on Asthma) guidelines, which would avoid the risk of potential DDI. In general, these guidelines are adhered to in the Caribbean, due to their high profile in public health campaigns on asthma [14].

While this study provides an important insight into the use of medication in patients in a low-and-middle-income country setting, there were a few limitations. As a single centre study, there was a risk of selection bias. However, Eric Williams Medical Sciences Complex serves approximately half of the population of Trinidad and Tobago, and census reports from the hospital suggest that the patients attending the ED are broadly representative of the ethnic and demographic make-up of the population as a whole. There was a risk of under-reporting of medication by study participants. While data was collected prospectively to minimise this, no attempt was made to verify patients’ accounts of their medication usage (such as checking drug dispensers or cross referencing with relatives and carers). Thus, we could not comment on whether patients were compliant with their medication, an important factor when considering potential DDI. In addition, Micromedex 2.0 did not list all the herbal medications found in our study, so any potential interactions involving these unlisted herbal remedies would have been missed. This may have contributed to the lack of association between herbal formulations and potential DDI, as may have the inadequate sample size of patients aged ≥65 years. This small sample size (which, for the older cohort of patients, was just under the calculated sample size of 276 patients) may also have contributed to some of the other statistically insignificant findings of this study, such as the lack of association between age, OTCs and potential drug–drug interactions.

As mentioned in the “Methods” section, this study focused on ED patients who were discharged from the department, as we were interested in the number of patients with potential DDIs who were being discharged with potentially no further follow up. However, the results do not tell us anything about the level of polypharmacy and potential DDIs in patients who were admitted. It may reasonably be surmised that these patients could have had a higher risk of polypharmacy and potential DDI as they were more unwell than those discharged home and therefore more likely to be on multiple medications.

As a result of our study, we intend to develop and assess programmes targeted at older ED attenders, including ED rounds with clinical pharmacists; implementation of a drug reconciliation service (involving the assessment of all patients aged ≥65 years on 5 or more medications by a clinical pharmacist and senior doctor prior to discharge); training doctors in safe prescribing practices in older patients and targeted health education campaigns.

## Conclusion

This study has identified a high level of polypharmacy and potential drug–drug interactions in patients attending the Emergency Department in Trinidad and Tobago, highlighting important risk factors that may predict these outcomes (including increasing age, chronic illnesses, and the use of herbal, combination and OTC remedies). Specifically, while OTC, increased age and herbal medications were all independently associated with an increased risk of polypharmacy, the only factors predictive of potential drug–drug interactions were polypharmacy and common chronic illnesses (diabetes, hypertension and ischaemic heart disease). Targeted initiatives aimed at older people and those with chronic illnesses are therefore likely to reduce the risk of polypharmacy and potential DDI in this setting.

## Electronic supplementary material

Below is the link to the electronic supplementary material.

Appendix 1: Herbal medication used by study participants (PNG 77 kb)

Appendix 2: Combination therapies used by study participants (PNG 67 kb)
