# Supplementary figures and images for: Polypharmacy and potential drug–drug interactions in emergency department patients in the Caribbean
Source: Int J Clin Pharm. 2017 Aug 9;39(5):1119–27. doi: 10.1007/s11096-017-0520-9 (PMC5686268; doi:10.1007/s11096-017-0520-9)

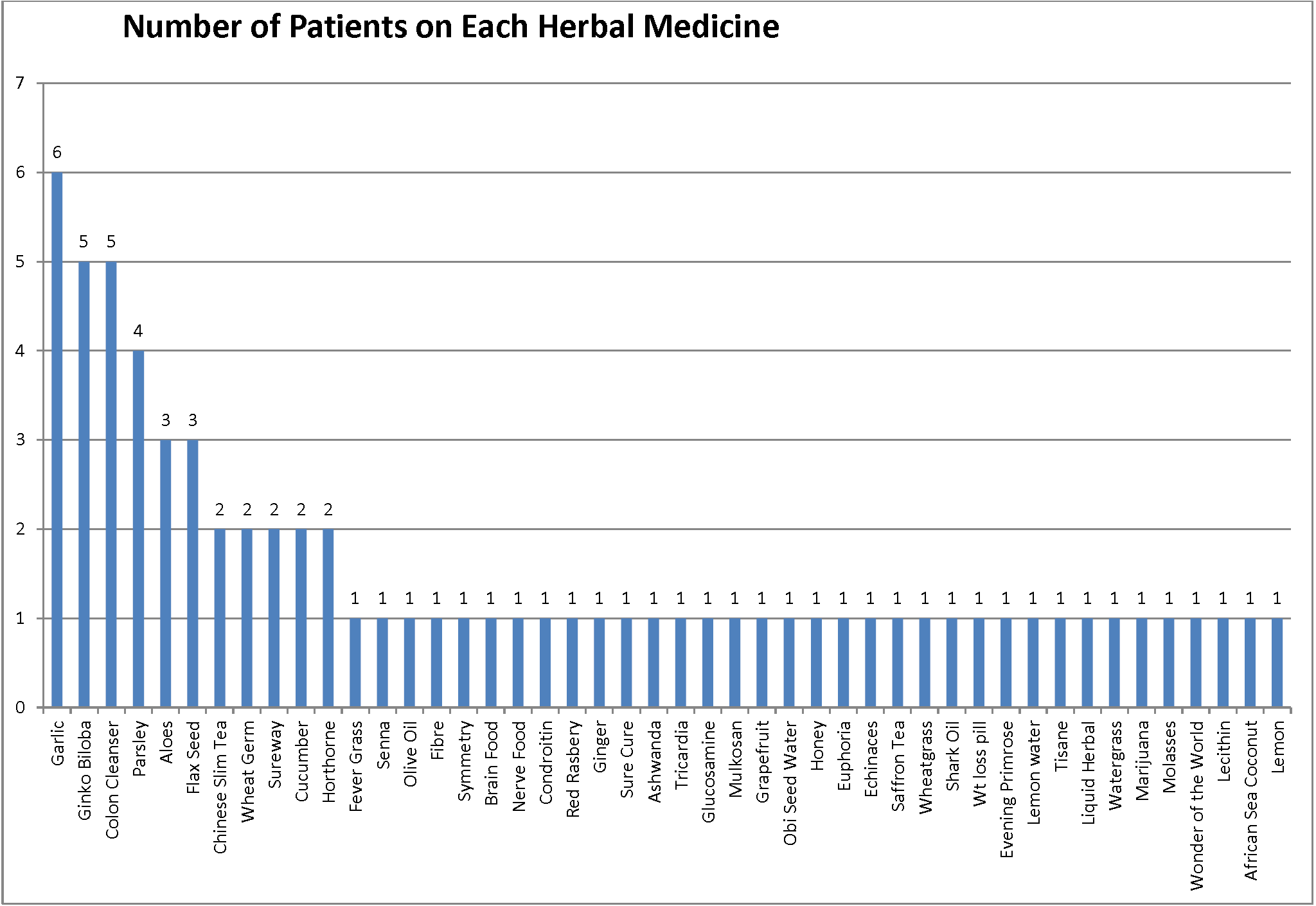

Supplement: Supplementary file 1 — Appendix 1: Herbal medication used by study participants (PNG 77 kb) [file 11096_2017_520_MOESM1_ESM.png]

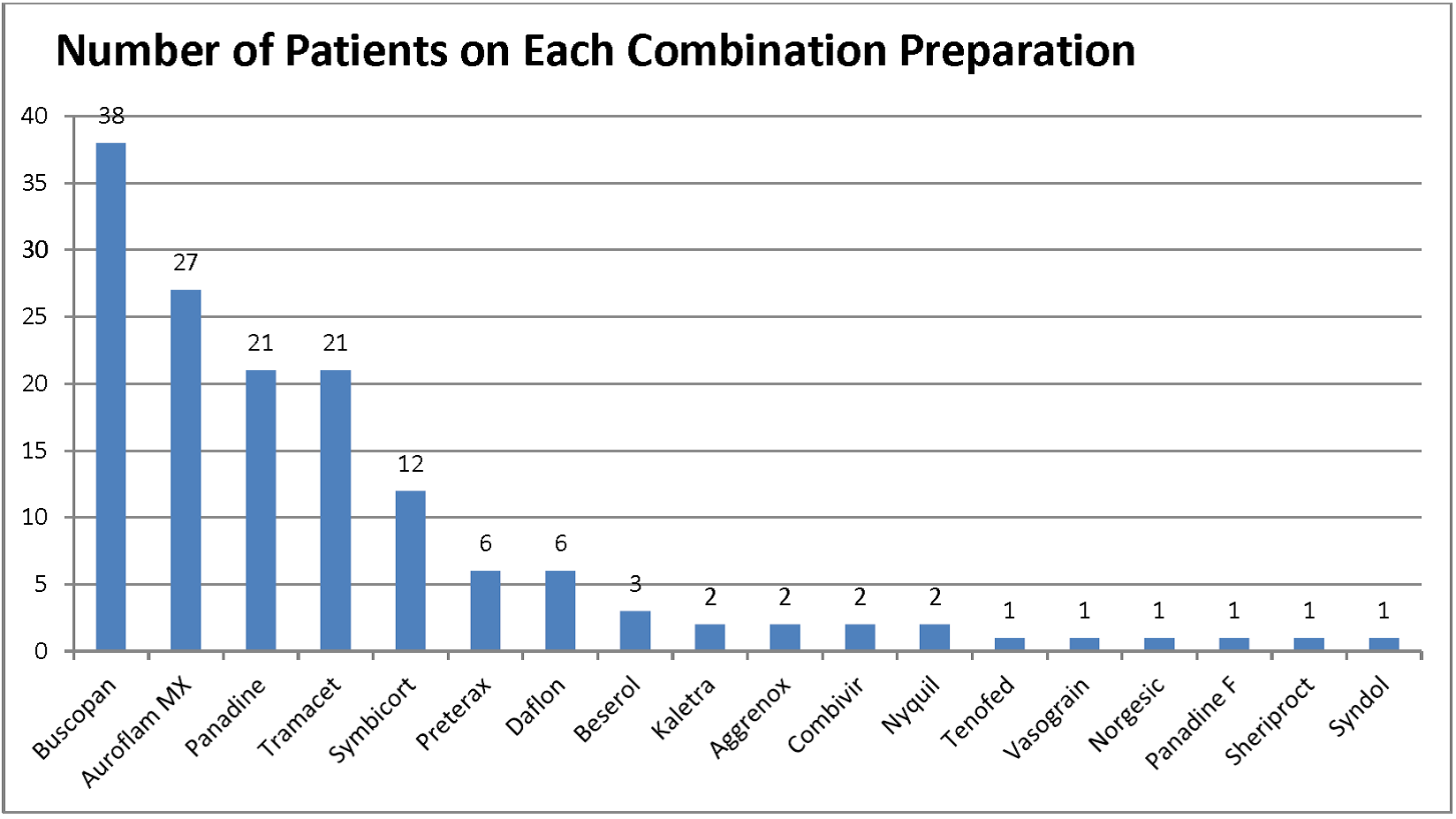

Supplement: Supplementary file 2 — Appendix 2: Combination therapies used by study participants (PNG 67 kb) [file 11096_2017_520_MOESM2_ESM.png]
